# Supplementary material for: The genetic architecture of temperature adaptation is shaped by population ancestry and not by selection regime
Source: Genome Biol. 2021 Jul 16;22:211. doi: 10.1186/s13059-021-02425-9 (PMC8285869; doi:10.1186/s13059-021-02425-9)
Supplement: Supplementary file 3 — Additional file 3. Review history. [file 13059_2021_2425_MOESM3_ESM.docx]

Review History

**First round of review**

**Reviewer 1**

**Are you able to assess all statistics in the manuscript, including the appropriateness of statistical tests used?** Yes, and I have assessed the statistics in my report.

**Comments to author:**

Comments

Scope and implications:
   
   The study by Otte et al. shows that the genetic architecture of thermal adaptation differs strongly (different haplotype structure) between two instances of experimental evolution in Drosofila simulans based on sampling of founder individuals from two different populations (Portugal and Florida) but under same hot treatment. A third instance, also from the Portugal population was under a cold treatment. The study shows that hot and cold adaptation in the Portugal lines had different targets of selection, with similar genetic architecture (similar haplotype structure).
   
   The conclusions of the study are that difference in the adaption architecture between populations is caused by difference in LD in the founder populations, causing difference in sampling of "ancestral" polymorphism. The difference between cold and hot adaptation is caused by the fact that adaptation to different selection regimes has a different genetic basis. Hot and cold should thus be seen as different traits.
   
   The main message is that polygenicity of quantitative traits poses a real challenge to the inference of the genetic basis of adaption, in ecological and human health contexts alike. This is an important point to make, and a valid reason to publish.

   The authors warn about any inference of the genetics of adaptation made from a single population, esp. in the context of climate adaptation and climate change.
   -    I think this is not an issue since most GEA (gene environment association) studies are based on sampling multiple populations on environmental gradients across space.
   
   The authors emphasize the polygenic nature of the traits studied, which would be the cause of lack of parallelism in the response to selection between founder populations.
   -   I think this is an important point to make although it is not a new insight. Previous studies have repeatedly pointed to that difficulty. However, it is not entirely clear to me whether polygenicity is the only cause here or variation in levels of adaptation among replicates could also explain the pattern. The present study cannot entirely answer that question.

   -   One of the major difficulty, and limitation of this study is the absence of fitness estimates (or of a phenotype, as noted by the authors). It is thus difficult to understand the pattern of adaptive genetic changes without an estimate of the level of adaptation achieved in the selected lines and founder populations. Only one sentence points to potential difference in fecundity/fitness between the two founder populations (line 302). Differences in prior adaptation to warm temperatures at the start of the experiments may explain part of the observed patterns. But how much? Also, not all replicates may have achieved similar levels of adaptation during the experiments but this hasn't been assessed either. In fact, we don't know whether there has been changes in average fitness in the selected lines at all during the 50 generations. Without such data, the validity of the underlying assumption of increase in fitness reached during this study cannot be assured. The only proof of adaptation is indirect, taken from the allele frequency changes. However, an independent validation of "adaptation" should be used to validate the pattern, otherwise the argument is circular: there has been adaptation because we observe allele frequency changes, and the allele frequency changes are adaptive because adaption occurred. I see this as a major weakness of the experimental design. The only strong argument in favour of adaptation is the parallelism of the allele frequency changes among replicates. While I do not strongly doubt of the actual evolution of the lines, the limitations of the study should be made clearer in the discussion. I see this as an essential missing part of the discussion.
   
   -   Lack of parallelism among replicates or experiments may be caused by the fact that strains have not reached the same level of adaptation (not same mean fitness) instead of evidence of genetic redundancy. Some strains may, for instance, remain closer to a local optimum different from a more global optimum reached in other strains. Or, because each strain has sampled a different set of starting alleles, their evolutionary dynamics may differ in mode (which allele is contributing to increase fitness) and tempo (time to fixation/rate of increase in frequency). Without individual or average strain fitness estimates, it will remain impossible to associate allele frequency, and change thereof with fitness. Any additional information on those aspects should be included in the study. If none available, implications and limitations should be clearly discussed.
   
   -   The text lacks clarity and consistency of the vocabulary used to designate key concepts like "contributing loci", "selection targets", phenotypic versus fitness effects, "genomic region" vs "haplotype block" etc., which in the long run confuses the reader and muddles the message. The text is also too long, and often redundant, should be shortened and streamlined.
   
   -   All this makes me conclude that the study lacks a strong, clearly articulated and validated take home message.
   
Presentation:

   I found the text at times convoluted and redundant, sometimes lacking consistency and clarity regarding concepts of selection and effect of alleles on a phenotypic trait under selection versus their resulting fitness effects. The text often mixes population genetics (fitness effect) and quantitative genetics (phenotypic effect) reasoning. The distinction between the genetic architecture and the adaptive architecture of a trait leads to some confusion, when it isn't clear which is the subject. Confusion comes from a lack of context implied by the term "contributing loci". Loci can contribute to a trait phenotype or a trait's variation (genetic architecture), or to adaptation of the population when responding to selection (adaptive architecture). Of course, the same loci contribute to both in principle, but the authors instate a distinction at the onset of the paper, which I think brings confusion throughout. In any case, the phrasing should be clarified as much as possible to avoid confusion.
   
   -   lines 31-33: what is the subject, genetic or adaptive architecture? although previous paragraph made a distinction, that distinction is lost in this paragraph because QTL/GWAS are mentioned before selection scans.
   
   -   lines 83-86: is the concept of "selected genomic regions" any different from "selection targets"? are selection targets reserved to shorter haplotypes/less loci under selection? can a genomic region harbour several selection targets? or any genomic region of any size can be a selection target? What is the relationship between all these terms?
       eg: lines 136-139: where we learn that a haplotype "block" contains one selection target but can carry more than one selection target.
             lines 232-233: "31 selected haplotype blocks..., which are considerably fewer selected alleles than the 88..." -> a block is an allele?  
   Lack of clarity and consistency in the vocabulary used is muddying the message. You should stick to a clear definition of the smallest detectable unit under selection, hopefully transferable between the two populations.
   
   -   parts of the discussion on whether cold and hot adaptation are different traits are somewhat redundant (sections p15 "Different trait optima..." & p22 "Temperature adaptation...", both also repeat arguments in the Results section)
   
   -   the long section on "Linkage disequilibrium" is interesting but requires mending of the text to streamline and avoid confusions:
       - line 347: favored loci -> favored alleles or contributing loci
       - lines 360, 368: do haplotypes have a fitness? they have an additive effect on the trait under stabilizing selection (from the simulation settings). However, whether that effect is beneficial or not depends on whether it brings the phenotype closer or farther from the trait optimum, which depends on the values at other haplotypes. There is thus context dependency (epistasis) for fitness. How does this come into play in the interpretation of the results? Please clarify.  
       - line 372: you mean 1000 contributing instead of 1200 (10x100 replicates)?
       - line 375: it is not clear how strong LD is
       - line 376: it is not clear what the phenotype is, how it is obtained and how it relates to fitness (or selection coefficient of a haplotype block).

Methodology, Results and interpretation:

   - p9: as acknowledged by the authors, the relationship between starting allele frequencies and selection coefficients is likely artefactual. The overall implications on detection of parallelism are not tested. That should be tested and discussed.
   -   I would also like to see a confirmation of the method bias with simulations (eg using the LD simulations).
   -   lines 208-210: the resemblance in number and distribution of selection coefficients is not that surprising if both analyses suffer from the same method bias. The possible method artifact should be ruled out first.
   
   - line 240, 250: effect of differing number of replicates on haplotype calling, is the difference in number of blocks artifactual? Do compare the results when using 5 replicates instead of 10 to see if the difference remains. This is done later to test for effect on selection coefficients and parallelism, but not for haplotype calling.
   - line 245-247: the reason for this correction is not clear to me, do bring the rational forth.
   
   - line 257 parallelism in what? It should made clear here that it is only based on binary present-absence data among replicates based on significant selection coefficients of selected alleles.
       - here again, it is not clear what an allele entails exactly, how is it defined?
   
   - lines 275-277: sentence starting with "As the frequency change..." very confusing. How can a haplotype be selected and not selected at the same time? How can a selected SNP be on a selected and non-selected haplotype at the same time? are we talking about natural selection? SNP/haplotype calling process? confusing...
   
   -    in general, please clarify the relationship between frequency change at SNP and a haplotype selection coefficient. Also define what is a "selected allele" relative to SNP frequencies and haplotype definition. These terms are used interchangeably without a clear hierarchy.

   
LD simulations:

- how strong was LD in the simulations? Was it similar to the observed LD? if not, why not?

- why compare LE with LD, when none of the founder population is at LE? The observed difference in LD is not that large, which may strongly weaken the difference in patterns observed from more realistic simulations. I would prefer to see an analysis of a case similar to the empirical data rather than one only remotely similar. I'd thus advise to run simulations with same difference in LD as estimated in the founder populations.

Methods, p30: the description of the simulation setting is incomplete and somewhat confusing. The following points need clarification:

   - specify the level of LD in the simulations, how it was achieved, and maintained (ie what was the recombination rate b/n loci within and among haplotypes)

   - how was the phenotypic trait modeled? were the loci diallelic or are you using a continuum-of-allele model? are mutations added to the existing allelic value at a locus, or do they replace the existing value? are the mutation effects drawn from a (normal) distribution or have fixed effects? what do you mean with "effect size of 0.05" -> is it the variance of the distribution of the mutational effects or the allelic value of the mutation? how is the phenotype determined from the genotype? is it assumed that causative loci contribute additively to the genotype and phenotype?

   - line 639: you mean SD of the fitness function, not of the phenotype

   - line 640: how can a mean fitness of -0.44 be reached when the minimum of the fitness function is 0.5?

   - line 640-41: what was the ancestral trait optimum?

   - line 644: what is a "favored allele"? from my understanding of the architecture of the trait under selection, loci are additive QTL, how would you then determine that an allele is favored versus disfavored? how would you calculate the selection coefficient of such an allele?

   - line 644, 649: what do you mean with "selected" or "favored" allele placed on only 200 chromosomes (individuals) in the starting population? what allele value was then used for the other 800?

   - are we right to assume that the population is a Wright-Fisher population with selection on female fecundity?

**Reviewer 2**

**Are you able to assess all statistics in the manuscript, including the appropriateness of statistical tests used?** Yes, and I have assessed the statistics in my report.

**Comments to author:**

Overall Review:  This is another commendable study from the Schloterer lab that combines next generation genome-wide sequencing of multiple evolving populations. It compares results obtained with two different selection regimes, one hot and one cold, and two different founding populations of Drosophila simulans, one from Portugal and one from Florida. The results reveal a lack of consistency among selection regimes and founding populations. Temperature-dependent adaptation is shown to be "local" or "contingent," rather than globally consistent. My chief recommendations for revision concern the experimental and theoretical contexts within which this study should be interpreted.
Major points with respect to revision:
1. I do not require any additional data collection or statistical revision. My comments are organized in terms of experimental paradigm (A) and theoretical significance (B).
A.
2. The typical paradigm for experimental evolution in this and many other labs is the use of hybrid populations created from isofemale lines. This method for founding experimental populations can be expected to generate some degree of random "historicity" as the isofemale lines approach fixation of specific genotypes with high stochasticity. Then there are further stochastic and historical effects which arise when such lines are hybridized. The strengths and limitations of this paradigm should be addressed. Most importantly, such stochastic historical effects might be the source of the discrepancy between the replicate lines derived from Portugal and Florida.
3. As the authors themselves point out, there is a comparative difficulty arising from the different scales of replication used for the Florida versus Portugal experiments. A further question for large-scale experimentation is whether a larger and uniform level of replication might produce more similar results, such as 20 evolving populations from each source. Or indeed, three sets of ten evolving populations from three distinct founders from the two sources, with a total of 60 evolving experimental lineages. The authors should discuss whether they believe that their findings would hold up with such an expanded experimental protocol.
3. The authors themselves point out at one point that there experiments might have benefitted from some phenotypic characterization of experimental adaptation. That raises the more general question of how the field of experimental evolution can progress with some labs just doing phenotypes and other labs just doing genome-wide sequencing. Surely this and other studies would be strengthened by combining the two types of assay. Furthermore, genomic evolution might be usefully analyzed in terms of the degree to which specific genomic changes can be associated with specific phenotypic changes, as that is surely the foundation for adaptation itself.
B.
4. This is yet another study that finds difficulties with the invocation of noroptimal selection along a one-dimensional functional gradient. Such models have been longstanding favorites of evolutionary geneticists, such as Kimura or Lande. But that preference may arise more from the ease with which they can be formulated and analyzed by theoreticians. Actual evolving populations don't seem to embody the percepts of such theory, whether the issue is the maintenance of genetic variation (see the work of Turelli, for example) or the evolution of closely studied populations, as illustrated by this study. An alternative point of view that the authors should discuss is that such models are indeed NOT usually correct.
5. There are alternative theoretical frameworks that address the maintenance of genetic variation and the response to selection. Among these are schemes of protected polymorphism arising from overdominance, epistasis, and frequency-dependent selection. If the thrust of the preceding point 4 is accepted, then it would be theoretically salient for the authors to suggest what type of underlying population genetic machinery would best account for their findings, in place of the noroptimal selection-mutation balance model.

**Authors Response**

**Point-by-point responses to the reviewers’ comments:**

Reviewer #1: Comments

Scope and implications:
The study by Otte et al. shows that the genetic architecture of thermal adaptation differs strongly (different haplotype structure) between two instances of experimental evolution in Drosofila simulans based on sampling of founder individuals from two different populations (Portugal and Florida) but under same hot treatment. A third instance, also from the Portugal population was under a cold treatment. The study shows that hot and cold adaptation in the Portugal lines had different targets of selection, with similar genetic architecture (similar haplotype structure).
The conclusions of the study are that difference in the adaption architecture between populations is caused by difference in LD in the founder populations, causing difference in sampling of "ancestral" polymorphism. The difference between cold and hot adaptation is caused by the fact that adaptation to different selection regimes has a different genetic basis. Hot and cold should thus be seen as different traits.
The main message is that polygenicity of quantitative traits poses a real challenge to the inference of the genetic basis of adaption, in ecological and human health contexts alike. This is an important point to make, and a valid reason to publish.

The authors warn about any inference of the genetics of adaptation made from a single population, esp. in the context of climate adaptation and climate change. - I think this is not an issue since most GEA (gene environment association) studies are based on sampling multiple populations on environmental gradients across space.

**We agree with the reviewer that some studies are using multiple samples distributed over a broad range of habitats. Nevertheless, this does not disqualify our point: if two populations are adapting to the same environment, but using different sets of genes, no association will be detected with the approaches aiming to correlate environmental variables with allele frequency differences. We were, however, referring to the most classic case that standard population genetic analyses are applied to a small number of populations relying only on the molecular signature.**

The authors emphasize the polygenic nature of the traits studied, which would be the cause of lack of parallelism in the response to selection between founder populations. - I think this is an important point to make although it is not a new insight. Previous studies have repeatedly pointed to that difficulty. However, it is not entirely clear to me whether polygenicity is the only cause here or variation in levels of adaptation among replicates could also explain the pattern. The present study cannot entirely answer that question.
- One of the major difficulty, and limitation of this study is the absence of fitness estimates (or of a phenotype, as noted by the authors). It is thus difficult to understand the pattern of adaptive genetic changes without an estimate of the level of adaptation achieved in the selected lines and founder populations. Only one sentence points to potential difference in fecundity/fitness between the two founder populations (line 302).

Differences in prior adaptation to warm temperatures at the start of the experiments may explain part of the observed patterns. But how much?

**We fully understand the concern of the reviewer, but since these fitness estimates were already published before, we did not emphasize them in this manuscript. We now also refer to the previously published fitness estimates in line 78-80 and provide a figure with the fitness estimates from individual replicate populations in the supplement as Supplementary Figure S1 and S2.**

Also, not all replicates may have achieved similar levels of adaptation during the experiments but this hasn't been assessed either. In fact, we don't know whether there has been changes in average fitness in the selected lines at all during the 50 generations. Without such data, the validity of the underlying assumption of increase in fitness reached during this study cannot be assured.

**The fitness estimates in Supplementary Fig. S1 and S2 show some variation among evolved replicates. Nevertheless, previous data analysis did not detect statistically significant differences between evolved replicates, but the difference between evolved and ancestral populations was significant (Mallard et al. 2018, Barghi et al.2019).**

The only proof of adaptation is indirect, taken from the allele frequency changes. However, an independent validation of "adaptation" should be used to validate the pattern, otherwise the argument is circular: there has been adaptation because we observe allele frequency changes, and the allele frequency changes are adaptive because adaption occurred. I see this as a major weakness of the experimental design. The only strong argument in favour of adaptation is the parallelism of the allele frequency changes among replicates. While I do not strongly doubt of the actual evolution of the lines, the limitations of the study should be made clearer in the discussion. I see this as an essential missing part of the discussion.

**We provide fitness estimates in the supplement (Supplementary Fig. S1 and S2).**

- Lack of parallelism among replicates or experiments may be caused by the fact that strains have not reached the same level of adaptation (not same mean fitness) instead of a more global optimum reached in other strains. Or, because each strain has sampled a different set of starting alleles, their evolutionary dynamics may differ in mode (which allele is contributing to increase fitness) and tempo (time to fixation/rate of increase in frequency). Without individual or average strain fitness estimates, it will remain impossible to associate allele frequency, and change thereof with fitness. Any additional information on those aspects should be included in the study. If none available, implications and limitations should be clearly discussed.

**Please see our response above-the differences between replicates were not significant.**

- The text lacks clarity and consistency of the vocabulary used to designate key concepts like "contributing loci", "selection targets", phenotypic versus fitness effects, "genomic region" vs "haplotype block" etc., which in the long run confuses the reader and muddles the message. The text is also too long, and often redundant, should be shortened and streamlined.

**We understand the concern of the reviewer, but the challenge is that our manuscript merges two worlds-quantitative genetics and molecular population genetics. Hence, we are facing the challenge to describe something abstract (i.e. selection target) which we cannot identify and the molecular signature we are using to make inferences about the selection targets (haplotype blocks). We have carefully revisited our nomenclatures and eliminated the term “selected allele”.**

**We also admit that the manuscript is not short, in particular the discussion is long, but we strongly feel that this length - even with some redundancy - is needed to allow a broader readership to follow the rather complex logic.**

-All this makes me conclude that the study lacks a strong, clearly articulated and validated take home message.

**Many thanks for the suggestion to make the take home message clearer, but we chose to not only highlight our preferred interpretation of the data, but at the same time also highlight that alternative interpretations may be possible.**

   Presentation:

   I found the text at times convoluted and redundant, sometimes lacking consistency and clarity regarding concepts of selection and effect of alleles on a phenotypic trait under selection versus their resulting fitness effects. The text often mixes population genetics (fitness effect) and quantitative genetics (phenotypic effect) reasoning. The distinction between the genetic architecture and the adaptive architecture of a trait leads to some confusion, when it isn't clear which is the subject. Confusion comes from a lack of context implied by the term "contributing loci". Loci can contribute to a trait phenotype or a trait's variation (genetic architecture), or to adaptation of the population when responding to selection (adaptive architecture). Of course, the same loci contribute to both in principle, but the authors instate a distinction at the onset of the paper, which I think brings confusion throughout. In any case, the phrasing should be clarified as much as
possible to avoid confusion.

**We have made some changes to the introduction to highlight that the focus of our study is the adaptive architecture.**

**We use the term "contributing loci" when we refer to loci, which are affecting the trait irrespective of whether they are responding to selection or not (hence we use "contributing loci" only in the context of genetic architecture, but not adaptive architecture). "Selection targets" is a generic term for loci or SNPs, which are responding to selection (i.e. a subset of the "contributing loci"). The molecular selection response is measured as selected "haplotype blocks", which change in frequency.**

**We went over the manuscript to assure a consistent use of the terminology, and we now provide a definition of these terms in Table 1.**

       -   lines 31-33: what is the subject, genetic or adaptive architecture? although previous paragraph made a distinction, that distinction is lost in this paragraph because QTL/GWAS are mentioned before selection scans.

**Has been corrected (line 27).**

       -   lines 83-86: is the concept of "selected genomic regions" any different from "selection targets"? are selection targets reserved to shorter haplotypes/less loci under selection? can a genomic region harbour several selection targets? or any genomic region of any size can be a selection target? What is the relationship between all these terms?         eg: lines 136-139: where we learn that a haplotype "block" contains one selection target but can carry more than one selection target.               lines 232-233: "31 selected haplotype blocks..., which are considerably fewer selected alleles than the 88..." -> a block is an allele?      Lack of clarity and consistency in the vocabulary used is muddying the message. You should stick to a clear definition of the smallest detectable unit under selection, hopefully transferable between the two populations.

**We have made some more efforts to standardize the terminology (see above), but some of the challenge comes from the fact that we cannot identify the causative SNPs and have to rely on genomic signature to make some inference and this is reflected in the nomenclature.**

       -   parts of the discussion on whether cold and hot adaptation are different traits are somewhat redundant (sections p15 "Different trait optima..." & p22 "Temperature adaptation...", both also repeat arguments in the Results section)

**This is correct, but we prefer to keep this redundancy, as it increases the readability of the manuscript.**

       -   the long section on "Linkage disequilibrium" is interesting but requires mending of the text to streamline and avoid confusions:

**We changed this part in line with our revised terminology to clarify the text (e.g. lines 345, 357, 370-374, 377, 413, 414).**

       - line 347: favored loci -> favored alleles or contributing loci

**We changed the text accordingly (line 345).**

       - lines 360, 368: do haplotypes have a fitness? they have an additive effect on the trait under stabilizing selection (from the simulation settings). However, whether that effect is beneficial or not depends on whether it brings the phenotype closer or farther from the trait optimum, which depends on the values at other haplotypes.

**Thanks for pointing this out-we replaced fitness with fitness effects (lines 358, 366).**

There is thus context dependency (epistasis) for fitness. How does this come into play in the interpretation of the results?**.** Please clarify.

**We agree with the reviewer that the epistasis for fitness is an important effect, but to what extent it comes into play depends on the distance to the trait optimum. We felt that an in depth discussion about dynamics when the trait optimum is being reached would add more complexity. We preferred not to expand on this-mainly because we only wanted to make the point that LD could have an effect on the selection response.**

        - line 372: you mean 1000 contributing instead of 1200 (10x100 replicates)?

**We are sorry for this misunderstanding and changed the text in line 375-376 to highlight that we simulated 10 contributing loci in a 1 Mb window. We also provide more information on this in the section about LD simulation methods (lines 638-669).**

       - line 375: it is not clear how strong LD is

**We provide now the average r^2^ estimates in Figure 4A.**

- line 376: it is not clear what the phenotype is, how it is obtained and how it relates to fitness (or selection coefficient of a haplotype block).

**We added the missing information on the relationship of genotype, phenotype and fitness in our LD simulations in line 370-374. In line with further comments of the reviewer, we also rewrote and extended our description of the LD simulations in the Material and Methods (lines 638-669) and included the requested information.**

Methodology, Results and interpretation:

   - p9: as acknowledged by the authors, the relationship between starting allele frequencies and selection coefficients is likely artefactual. The overall implications on detection of parallelism are not tested. That should be tested and discussed.

**We do not agree with the reviewer that the correlation is likely to be artifactual, as it is predicted and has been previously shown [1]. Nevertheless, we felt that it is important to highlight a potential complication.**

   -   I would also like to see a confirmation of the method bias with simulations (eg using the LD simulations).
   -   lines 208-210: the resemblance in number and distribution of selection coefficients is not that surprising if both analyses suffer from the same method bias. The possible method artifact should be ruled out first.

**We understand the concern of the reviewer, but it is not clear to us which additional insights can be gained from simulations, as it is obvious that low frequency alleles with a small selection coefficient are more difficult to detect. The challenge is whether this is sufficient to explain the observed correlation. To address this we would need to know the frequency of the selected loci and their effect size distribution, two key parameters that are not known and need to be investigated.**

       - line 240, 250: effect of differing number of replicates on haplotype calling, is the difference in number of blocks artifactual? Do compare the results when using 5 replicates instead of 10 to see if the difference remains. This is done later to test for effect on selection coefficients and parallelism, but not for haplotype calling.

**The reviewer is right that we did not explore the identification of haplotype blocks based on five replicates only. Now, we also tested the effect of replication on haplotype reconstruction and included our results in lines 614-619. Most interestingly, we detected only few haplotypes in subsets of five replicates in the Florida population. We attribute this to the high redundancy and thus few haplotype blocks rising in parallel, which reduces the power to detect SNPs with parallel frequency trajectories. Hence, we conclude that this is another indicator of lower effect sizes and a less parallel signal between replicates in Florida compared to the Portugal population.**

   - line 245-247: the reason for this correction is not clear to me, do bring the rational forth.

**The allele frequency change depends on the frequency: even if the effect size is the same, alleles closer to 0.5 experience more frequency change than alleles with a lower frequency.**

       - line 257 parallelism in what? It should made clear here that it is only based on binary present-absence data among replicates based on significant selection coefficients of selected alleles.

**We address this comment in line 254-256.**

       - here again, it is not clear what an allele entails exactly, how is it defined?

**The unit of interest is the selected haplotype block. We rewrote this part for clarification line 254-256.**

       - lines 275-277: sentence starting with "As the frequency change..." very confusing. How can a haplotype be selected and not selected at the same time? How can a selected SNP be on a selected and non-selected haplotype at the same time? are we talking about natural selection? SNP/haplotype calling process? confusing...

**We think that the confusion arises from the difference between selected SNPs and SNPs that are located on the same haplotype as the selected SNP. We are referring to the case in which a SNP is located on the same haplotype block as the selected SNP. Importantly, this linked SNP may not be in full LD with the selected SNP. Hence, it is also present on other non-selected haplotypes.**

**If we consider this partially linked SNP in the two populations, the frequency of this SNP on the non-selected haplotypes may differ between populations. Or in other words, the extent of LD could differ between the two populations. A consequence of low LD is that the frequency change of this SNP will not be highly correlated with the selected SNP, and therefore, it is not included in the set of marker SNPs of this population (but in the other population it could be part of the marker SNP set).**

**We have rephrased the text in line 273-281 to make this clearer.**

       -    in general, please clarify the relationship between frequency change at SNP and a haplotype selection coefficient. Also define what is a "selected allele" relative to SNP frequencies and haplotype definition. These terms are used interchangeably without a clear hierarchy.

**The marker SNPs are used to estimate the frequency of the selected haplotype block, and the frequency change of this haplotype block is used to estimate the selection coefficient. In the wake of the revised terminology we removed the term “selected allele”.**

   LD simulations:

- how strong was LD in the simulations? Was it similar to the observed LD? if not, why not?

**We thank the reviewer for this remark and included the distribution of average r^2^ estimates per simulation and scenario in Figure 4A. Unfortunately, we do not have realistic estimates for LD between the selected SNPs in our experiments, as the actual selected SNPs are not known. Although genome-wide long-range LD estimates as depicted in Figure 5 indicate a general pattern of higher LD in the Portugal population, it did not provide realistic estimates for LD among selection targets. We therefore chose a qualitative proof-of-principle approach to examine the effect of LD and LE in one genomic region.**

- why compare LE with LD, when none of the founder population is at LE? The observed difference in LD is not that large, which may strongly weaken the difference in patterns observed from more realistic simulations. I would prefer to see an analysis of a case similar to the empirical data rather than one only remotely similar. I'd thus advise to run simulations with same difference in LD as estimated in the founder populations.

**Please see above.**

Methods, p30: the description of the simulation setting is incomplete and somewhat confusing. The following points need clarification:

**We thank the reviewer for the helpful questions and remarks which helped to clarify our simulations, both, rationale and procedure.**

   - specify the level of LD in the simulations, how it was achieved, and maintained (ie what was the recombination rate b/n loci within and among haplotypes)

**We included the actual r^2^ estimates in Figure 4A. In addition, we now describe our procedure for achieving high LD and linkage equilibrium in more detail (lines 662-669) and include the recombination rate estimate for *Drosophila simulans* which we used for all simulations in lines 656-658.**

    - how was the phenotypic trait modeled? were the loci diallelic or are you using a continuum-of-allele model? are mutations added to the existing allelic value at a locus, or do they replace the existing value? are the mutation effects drawn from a (normal) distribution or have fixed effects? what do you mean with "effect size of 0.05" -> is it the variance of the distribution of the mutational effects or the allelic value of the mutation? how is the phenotype determined from the genotype? is it assumed that causative loci contribute additively to the genotype and phenotype?

**We thank the reviewer for these comments and included the missing information in the LD simulation method section (lines 641-658). We modeled diallelic loci and did not include *de novo* mutations in our simulations, as adaptation in a typical *Drosophila* Evolve and Resequence setting mainly occurs from standing genetic variation [2].**

   - line 639: you mean SD of the fitness function, not of the phenotype

**Now we clarify that our Gaussian fitness function was defined as N(0.5;1.2) scaled to a maximum of 4.5 and a minimum of 0.5 (lines 652-654).**

   - line 640: how can a mean fitness of -0.44 be reached when the minimum of the fitness function is 0.5?

**Thanks for spotting this mistake, we rewrote this part and included the correct estimates for average genotypic value, phenotypic value and fitness of the ancestral population in lines 654-656.**

   - line 640-41: what was the ancestral trait optimum?

**We include information on the ancestral population in lines 654-656.**

   - line 644: what is a "favored allele"? from my understanding of the architecture of the trait under selection, loci are additive QTL, how would you then determine that an allele is favored versus disfavored? how would you calculate the selection coefficient of such an allele?

**We have rewritten this section to make it clearer. As we modeled our contributing loci as diallelic, one allele contributed to the quantitative trait under selection based on its positive effect size while the other allele has a negative effect on the trait. As we modeled a quantitative trait, we did not calculate selection coefficients for these alleles.**

   - line 644, 649: what do you mean with "selected" or "favored" allele placed on only 200 chromosomes (individuals) in the starting population? what allele value was then used for the other 800?

**We rewrote lines 660-662 to clarify that we generated chromosomes containing either the allele contributing to the quantitative trait under selection with a positive effect size, or the allele having a negative effect on the trait.**

   - are we right to assume that the population is a Wright-Fisher population with selection on female fecundity?

**We thank the reviewer for this comment and added the information on our simulated population being a Wright-Fisher population with selection in lines 639-642. However, we did not include sex-specific selection in our simulations, therefore selection acted on both, male and female fecundity equally.**

Reviewer #2: Overall Review:  This is another commendable study from the Schloterer lab that combines next generation genome-wide sequencing of multiple evolving populations. It compares results obtained with two different selection regimes, one hot and one cold, and two different founding populations of Drosophila simulans, one from Portugal and one from Florida. The results reveal a lack of consistency among selection regimes and founding populations. Temperature-dependent adaptation is shown to be "local" or "contingent," rather than globally consistent. My chief recommendations for revision concern the experimental and theoretical contexts within which this study should be interpreted.
Major points with respect to revision:
1. I do not require any additional data collection or statistical revision. My comments are organized in terms of experimental paradigm (A) and theoretical significance (B).
A.
2. The typical paradigm for experimental evolution in this and many other labs is the use of hybrid populations created from isofemale lines. This method for founding experimental populations can be expected to generate some degree of random "historicity" as the isofemale lines approach fixation of specific genotypes with high stochasticity. Then there are further stochastic and historical effects which arise when such lines are hybridized. The strengths and limitations of this paradigm should be addressed. Most importantly, such stochastic historical effects might be the source of the discrepancy between the replicate lines derived from Portugal and Florida.

**The experiments were started from freshly established isofemale lines, that were collected from the same population. Hence, we do not consider this a hybrid population, but a representative sample of a natural population. We do not think that we have introduced a bias during the sampling that could have affected the results. Rather, we think that the differences we observe reflect the actual history of the natural populations.**

3. As the authors themselves point out, there is a comparative difficulty arising from the different scales of replication used for the Florida versus Portugal experiments. A further question for large-scale experimentation is whether a larger and uniform level of replication might produce more similar results, such as 20 evolving populations from each source. Or indeed, three sets of ten evolving populations from three distinct founders from the two sources, with a total of 60 evolving experimental lineages. The authors should discuss whether they believe that their findings would hold up with such an expanded experimental protocol.

**We tested for an influence of different numbers of replicates by down-sampling the number of replicates from the Florida population and found that this cannot explain the differences we have seen.
Increasing the number of replicates would probably have detected more realizations of the same polygenic adaptation. Since we find it too speculative to talk about the impact of more replicates, we prefer not to discuss this in the manuscript.**

3. The authors themselves point out at one point that there experiments might have benefitted from some phenotypic characterization of experimental adaptation. That raises the more general question of how the field of experimental evolution can progress with some labs just doing phenotypes and other labs just doing genome-wide sequencing. Surely this and other studies would be strengthened by combining the two types of assay. Furthermore, genomic evolution might be usefully analyzed in terms of the degree to which specific genomic changes can be associated with specific phenotypic changes, as that is surely the foundation for adaptation itself.

**The reviewer raises a super interesting and important point, which uncovers probably the greatest weakness of the *Drosophila* system.**

1) **We do not have a good understanding on which phenotypes to study: Kellermann et al [3] studied phenotypes classically associated with clinal (and hence temperature) variation, but did not find a consistent response when selecting for temperature. A similar pattern was seen in a study from our lab [4]. Hence, following these phenotypes would not have led to a better understanding of adaptation.**
2) **Nevertheless, we have demonstrated on several occasions [1, 5-7] that based on the evolved populations, it is possible to identify interesting phenotypes, which most likely were responsible in the adaptation process. It would be great if we could go back to stocks frozen during the experiment and to measure these phenotypes during the experiment-but this is not possible in fruit flies.**
3) **We would like to point out that most phenotype based work in experimental evolution does not employ the same selection regime as our study. While we used laboratory natural selection (i.e. we changed the environment and measure the selection response), the other studies use truncating selection. Nevertheless, the allele frequency trajectories (and phenotypic evolution) will be quite different-in particular when selection is continued for many generations.**

B.
4. This is yet another study that finds difficulties with the invocation of noroptimal selection along a one-dimensional functional gradient. Such models have been longstanding favorites of evolutionary geneticists, such as Kimura or Lande. But that preference may arise more from the ease with which they can be formulated and analyzed by theoreticians. Actual evolving populations don't seem to embody the percepts of such theory, whether the issue is the maintenance of genetic variation (see the work of Turelli, for example) or the evolution of closely studied populations, as illustrated by this study. An alternative point of view that the authors should discuss is that such models are indeed NOT usually correct. 5. There are alternative theoretical frameworks that address the maintenance of genetic variation and the response to selection. Among these are schemes of protected polymorphism arising from overdominance, epistasis, and frequency-dependent selection. If the thrust of the preceding point 4 is accepted, then it would be theoretically salient for the authors to suggest what type of underlying population genetic machinery would best account for their findings, in place of the noroptimal selection-mutation balance model.

**We agree with the reviewer that our data suggest that the widely used model is probably not well-suited to explain our data. Nevertheless, we do not think that our data are putting us in a position to propose and test an alternative model. We are convinced that our manuscript provides an important step to initiate some scrutinizing of the old models, but proposing a new one is clearly beyond the scope of this manuscript.**

**References**

1.	Barghi N, Tobler R, Nolte V, Jaksic AM, Mallard F, Otte KA, Dolezal M, Taus T, Kofler R, Schlötterer C: **Genetic redundancy fuels polygenic adaptation in *Drosophila*.** *PLoS Biol* 2019, **17:**e3000128.

2.	Burke MK, Dunham JP, Shahrestani P, Thornton KR, Rose MR, Long AD: **Genome-wide analysis of a long-term evolution experiment with *Drosophila*.** *Nature* 2010, **467:**587-590.

3.	Kellermann V, Hoffmann AA, Kristensen TN, Moghadam NN, Loeschcke V: **Experimental evolution under fluctuating thermal conditions does not reproduce patterns of adaptive clinal differentiation in *Drosophila melanogaster*.** *American Naturalist* 2015, **186:**582-593.

4.	Tobler R, Hermisson J, Schlötterer C: **Parallel trait adaptation across opposing thermal environments in experimental *Drosophila melanogaster* populations.** *Evolution* 2015, **69:**1745-1759.

5.	Mallard F, Nolte V, Tobler R, Kapun M, Schlötterer C: **A simple genetic basis of adaptation to a novel thermal environment results in complex metabolic rewiring in *Drosophila*.** *Genome Biol* 2018, **19:**119.

6.	Hsu SK, Jaksic AM, Nolte V, Lirakis M, Kofler R, Barghi N, Versace E, Schlötterer C: **Rapid sex-specific adaptation to high temperature in *Drosophila*.** *Elife* 2020, **9**.

7.	Jaksic AM, Karner J, Nolte V, Hsu SK, Barghi N, Mallard F, Otte KA, Svecnjak L, Senti KA, Schlötterer C: **Neuronal function and dopamine signaling evolve at high temperature in Drosophila.** *Mol Biol Evol* 2020.

**Second round of review**

**Reviewer 1**

I appreciate the efforts made by the authors to provide more information showing the fitness differences between experimental replicates of the evolved lines, and to have clarified the presentation of the results. The addition of glossary box greatly helps in that respect.
